# Investigating BLS instructors’ ability to evaluate CPR performance: focus on compression depth, rate, and recoil

**DOI:** 10.1186/s12873-024-01162-z

**Published:** 2025-01-29

**Authors:** Shih-Jhan Lin, Chih-Jan Chang, Shao-Chung Chu, Ying-Hsin Chang, Ming-Yuan Hong, Po-Chang Huang, Chia-Lung Kao, Chih-Hsien Chi

**Affiliations:** 1 Department of Emergency Medicine, College of Medicine, National Cheng Kung University Hospital, National Cheng Kung University, No.138, Sheng Li Road, Tainan city, 704 Taiwan; 2 Taiwan Medical Device Innovation Center, National Cheng Kung University, Tainan, Taiwan

**Keywords:** Basic Life Support instructors, CPR quality assessments, Correlation analysis

## Abstract

**Background:**

Out-of-hospital cardiac arrest (OHCA) presents significant challenges with low survival rates, emphasizing the need for effective bystander CPR training. In Basic Life Support (BLS) training, the role of instructors is pivotal as they assess and correct learners’ cardiopulmonary resuscitation (CPR) techniques to ensure proficiency in life-saving skills. This study evaluates the concordance between CPR quality assessments by Basic Life Support (BLS) instructors and those determined through Quantitative CPR (QCPR) devices, utilizing data from BLS courses conducted at National Cheng Kung University Hospital from October 2017 to April 2018.

**Methods:**

The study analyzed existing data from BLS courses, comparing CPR quality assessments made by instructors with those recorded by QCPR devices. Key metrics such as chest compression speed, depth, and recoil were examined to identify the degree of consistency between human and automated evaluations.

**Results:**

In this study, CPR performance was analyzed using QCPR devices and BLS instructors across metrics like speed, depth, and recoil. Employing the Cohen kappa statistic revealed moderate to low interrater reliability, the kappa value is 0.65 (95% C.I. 0.65–0.65) for depth, 0.56 (95% C.I. 0.33–0.79) for speed, and 0.50 (95% C.I.0.28–0.71) for recoil. Correlation analysis visualized in a heatmap indicated a higher consistency in depth evaluations (correlation coefficient = 0.7) compared to speed and recoil, suggesting a need for improved alignment in CPR training assessments.

**Conclusions:**

The study underscores the importance of refining CPR training methods and adopting advanced technological aids to enhance the reliability of CPR skill assessments. By improving the accuracy of these evaluations, the training can be better tailored to increase the effectiveness of life-saving interventions, potentially boosting survival rates in out-of-hospital cardiac arrest scenarios.

## Introduction

Out-of-hospital cardiac arrest (OHCA) presents a critical public health challenge characterized by alarmingly low survival rates—approximately 10% [1]. The urgency of enhancing survival outcomes has led to a significant emphasis on the quality of immediate care provided by bystanders. Research has shown that high-quality cardiopulmonary resuscitation (CPR) performed by bystanders can significantly increase the likelihood of survival following cardiac arrest [2]. In response to this need, resuscitation guidelines, particularly those encapsulated in the 2015 Basic Life Support (BLS) guidelines, have been simplified. These guidelines advocate that chest compressions be between 2 and 2.5 inches (5 to 6 cm) deep at a rate of 100 to 120 compressions per minute, with careful attention given to allowing the chest wall to return to its natural position between compressions [3].

The accessibility and simplicity of these guidelines are critical, as they are primarily targeted at laypersons, who are often the first responders in such emergencies. A significant number of these laypersons acquire CPR skills through BLS courses, which also cover the use of automated external defibrillators (AEDs). In these courses, instructors play a pivotal role in ensuring that the correct techniques are communicated and demonstrated effectively. However, research indicates potential gaps in the capabilities of some instructors, particularly in accurately assessing CPR skills. The issues noted include frequent false-positive assessments of compression depth, which could undermine the effectiveness of the training [4–6].

Evaluating CPR performance, especially in terms of compression depth, rate, and the adequacy of chest wall recoil, involves visual assessments in which recognition of chest compression depth can be closely associated with the compression rate. Misidentification of adequate chest compression depth as deep increases as the compression rate increases [7]. This complexity necessitates a closer examination of the assessment skills of BLS instructors to ensure that they can reliably identify both adequate and inadequate CPR performance. In addition, peer feedback was considered feasible and useful for improving tutors’ facilitation skills [8].

Improving the overall quality of CPR training provided to laypersons potentially increases survival rates in cases of OHCA [9]. This study evaluates the ability of certified BLS instructors to accurately assess key CPR parameters—compression speed, depth, and recoil—using a CPR feedback device. Research indicates that such devices improve CPR skills acquisition and retention, making their integration into training and clinical practice valuable [10]. By examining instructor assessment capabilities, the study aims to pinpoint areas for improvement and recommend targeted interventions to enhance CPR quality.

## Methods

### Study design

The objective of this study was to conduct a consistency assessment of data from manikin studies across several BLS provider courses. This analysis compared the CPR quality evaluations made by instructors with those recorded by manikins. The aim was to identify any discrepancies and alignments in CPR quality assessments between human instructors and automated manikin feedback, ultimately enhancing the effectiveness of BLS training. Ethical approval was obtained from the ethics review board of National Cheng Kung University Hospital. (IRB: NCKUH B-EX-113-014).

### Participants

The participants for this study were selected from BLS provider courses held at National Cheng Kung University Hospital from October 1, 2017, to April 30, 2018. The cohort comprised 107 student learners in ACLS course, including medical staff, social workers, and members of the general public, representing a broad spectrum of backgrounds.

In addition, 15 seasoned BLS instructors were recruited to assess CPR performance throughout the study. These instructors predominantly had significant experience, the teaching experience for the 15 BLS instructors was a median of 60 (IQR 36, 180) months. Prior to leading hospital-wide BLS training sessions, all instructors underwent a standard training course to refine their skills in accurately assessing CPR quality.

### Study protocol

**Fig. 1:**
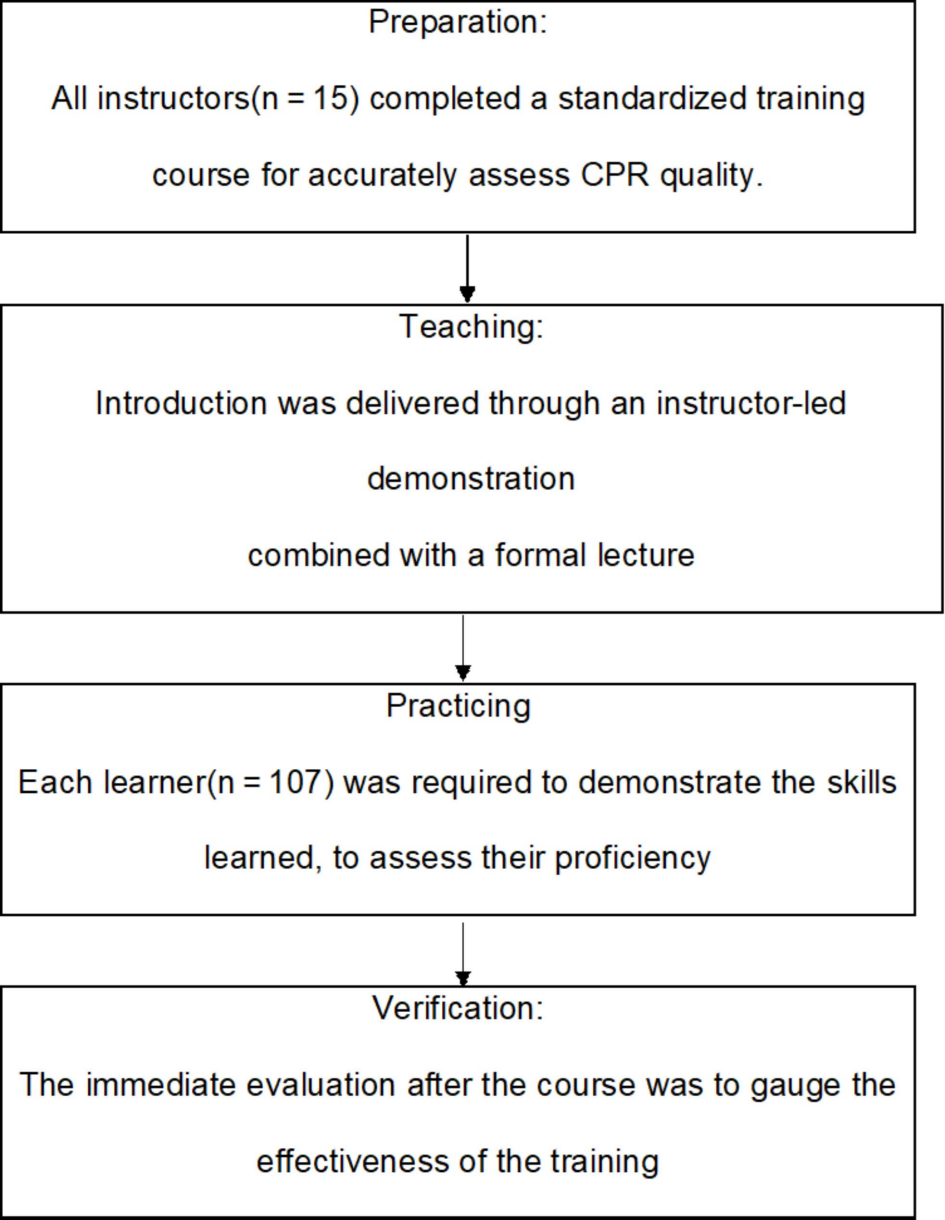
Organization of BLS provider course

The organization of BLS provider course is described in Fig. 1. The BLS training session closely adhered to the European Resuscitation Council (ERC) course structure. It commenced with an introductory segment lasting 30 min, during which BLS skills were presented to all the learners. This introduction was delivered through an instructor-led demonstration combined with a formal lecture; both formats meticulously followed the guidelines set by the ERC. After the initial presentation, the learners were required to actively engage by guiding the instructor through the skills step by step, ensuring a thorough understanding and practical application of the BLS procedures.

Following the guided practice, each learner was required to demonstrate the skills learned, to assess their proficiency. Each participant was evaluated right after the course to assess the effectiveness of the training and retention of the skills taught.’

For the practical assessment of BLS skills, Laerdal Resusci Anne manikins, positioned on the floor to simulate real-life scenarios, were used. Throughout the study period, 107 participants were meticulously observed performing CPR by 15 BLS instructors using four manikins per course. The instructors focused their evaluations on three cycles of chest compressions as part of the BLS procedure, which is critical for assessing the learners’ ability to perform essential lifesaving techniques. To maintain standardization and mitigate any potential biases, a research assistant closely monitored the sessions. This oversight ensured that the instructors had no access to the CPR quality data collected by the manikin and remained unaware of any feedback provided by the manikin during both the training and skill assessment phases. Each instructor was responsible for independently completing a BLS assessment form for every participant, documenting their observations and learner performance.

### Measures

Resusci Anne, produced by Laerdal in Wappingers Falls, NY, is used in CPR training, often connected to the SkillReporter system for performance tracking [11, 12]. The system captures comprehensive performance data which includes several key metrics: (1) the correct positioning of hands, (2) the rate of compressions (3) the depth of each compression (4) the completeness of chest recoil between compressions. The manikin features chest characteristics that are more lifelike, enhancing its utility for simulating clinical conditions. It has become a vital resource for both CPR research and training programs [13, 14]. This detailed tracking helps instructors provide precise, targeted feedback to improve the effectiveness of trainees’ CPR techniques.

We use a detailed rating scale to evaluate each subject’s CPR performance, focusing on three essential parameters: depth, speed, and recoil. This scale is structured into five distinct levels, each reflecting a specific range of performance accuracy. We used a 5-point scale to evaluate the BLS instructors in our training courses. For consistency and convenience, the same 5-point scoring scale was applied to the SkillReporter system to test the agreement between the two systems. Accordingly, the SkillReporter system provides percentile rank feedback, evaluating the overall correctness of the chest compression process. This approach allows for precise and detailed feedback on each component of the CPR technique. For example, achieving 0–20% accuracy in the performance skill earns a score of 1, 21–40% earns 2 points, 41–60% earns 3 points, 61–80% earns 4 points, and 81–100% accuracy earns 5 points. To ensure consistency in our analysis, we also convert the performance data obtained from manikins into scores that correspond to this same scale. This approach allows for a direct comparison and thorough analysis of the consistency between the assessments made by instructors and the automated data from manikins. It provides a clear metric of agreement and identifies areas where the accuracy of CPR training could be improved.

### Data analysis

To assess the consistency between the BLSI and QCPR on evaluating the quality of the chest compressions, Cohen’s kappa [15] was applied on the data analysis instead of applying Fleiss’ kappa. Fleiss’ kappa is suitable for analyzing the agreement among three or more raters, not for comparing two systems in the study design. Additionally, we utilized correlation coefficients to create a correlation heatmap that illustrates the relationships between individual metrics. IBM SPSS statistics 20 was used to analyze the data.

## Results

In this study, Tables 1, 2 and 3 detail the evaluations of subjects’ CPR performance by QCPR devices and BLS instructors, with a focus on speed, depth, and recoil metrics. To assess the alignment between these two evaluation methods, the Cohen kappa statistic was employed, providing a measure of interrater reliability for the different CPR performance indicators. (Table 4)

**Table 1:** Modified scale of speed distribution

Modified speed scale		QCPR				
		1	2	3	4	5
BLSI	1	9	2	1	1	0
	2	3	0	4	1	0
	3	2	2	1	5	5
	4	6	5	8	11	14
	5	1	0	2	7	17

**Table 2:** Modified scale of depth distribution

Modified depth scale		QCPR				
		1	2	3	4	5
BLSI	1	6	0	0	0	0
	2	3	0	0	1	0
	3	6	6	3	4	3
	4	1	2	3	10	13
	5	0	4	0	10	32

**Table 3:** Modified scale of recoil distribution

Modified recoil scale		QCPR				
		1	2	3	4	5
BLSI	1	1	0	1	0	1
	2	2	2	3	2	1
	3	7	3	3	3	2
	4	0	3	7	13	16
	5	1	1	2	7	26

**Table 4:** Cohen’s Kappa value between BLSI and QCPR

	Kappa value (95% confidence interval)	Level of agreement^*^
Speed	0.56(0.33–0.79)	Weak
Depth	0.65(0.65–0.65)	Moderate
Chest recoil	0.50(0.28–0.71)	Weak

^*^ To evaluate the agreement between two systems, the value of Kappa above 0.90 is suggested as “Almost perfect”, value between 0.80–0.90 is suggested as “Strong”, the value ranged between 0.70–0.79 is suggested as “Moderate”, the value between 0.40–0.59 is suggested as “Weak”, the value between 0.21–0.39 is suggested as “Minimal”, and the value ranged between 0-0.20 is suggested as “None” [15].

The analysis results show varying levels of agreement between the assessments made by BLS instructors and those recorded by QCPR devices. Specifically, the weighted kappa value for chest compression depth achieved a moderate agreement score of 0.65, indicating a relatively high level of consistency in this area. In contrast, the scores for speed and recoil showed lower levels of agreement, with weighted kappa values of 0.56 and 0.50, respectively.

**Fig. 2:**
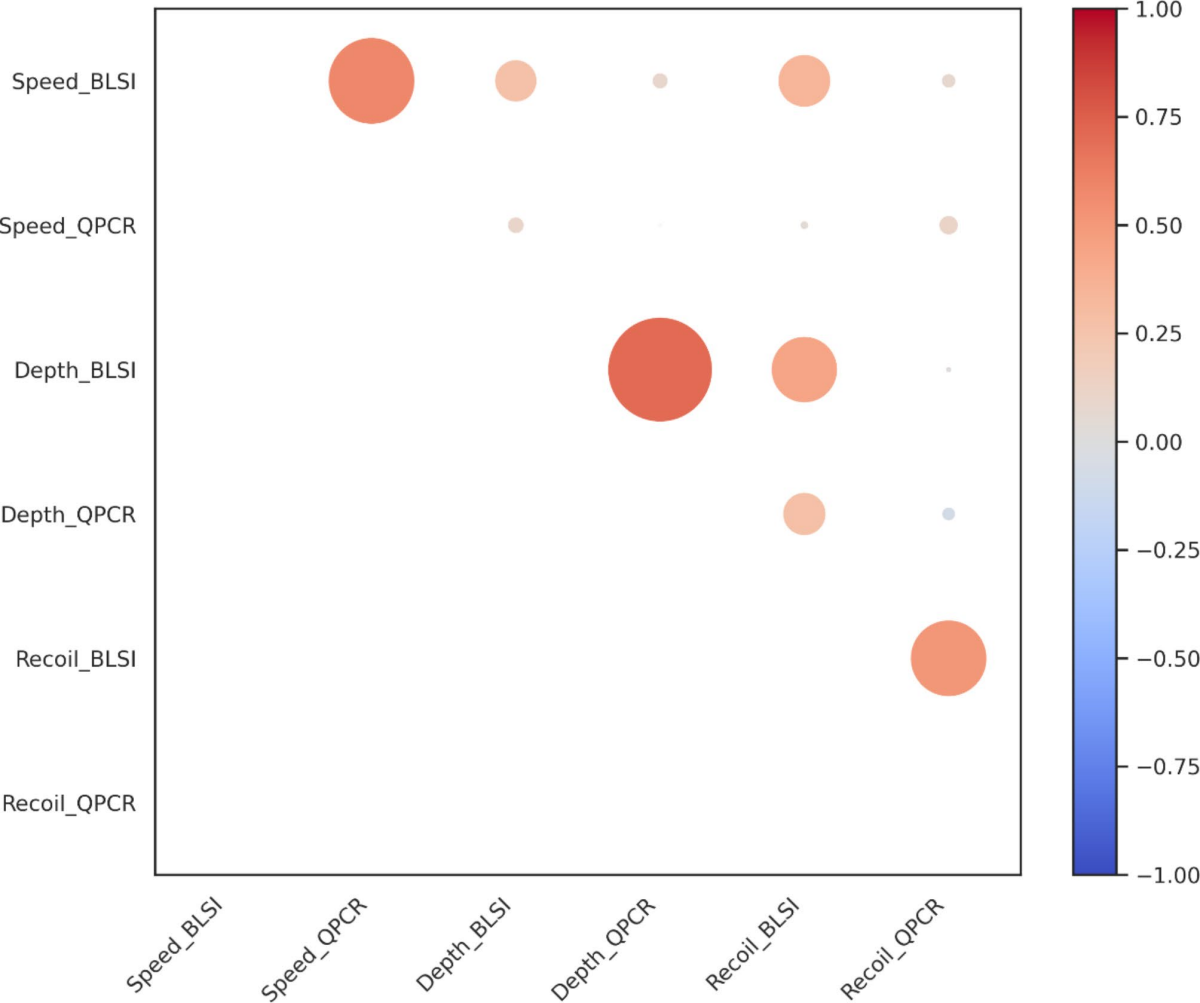
Heatmap of the Correlation between QPCR and BLSI Red indicates positive correlation coefficients, while blue represents negative correlations. The intensity of the color reflects the strength of the correlation between the measured variables, and the size of the circles corresponds to the magnitude of these correlations

In our study, we conducted a correlation coefficient analysis on the six categories for chest compression speed, depth, and recoil, as evaluated by both BLS instructors and QCPR devices. The results of these analyses were visualized via a heatmap to illustrate the relationships more clearly. Notably, the correlation coefficient for the depth scores between the BLS instructors and the QCPR was found to be 0.7. In contrast, the scores for speed and recoil showed lower correlations, at 0.58 and 0.51, respectively. (Fig. 2)

Moreover, the analysis revealed that the correlation coefficients within the group of BLS instructors were generally greater than those observed within the QCPR group.

## Discussion

This study reveals significant discrepancies in how certified BLS instructors evaluate CPR skills, specifically, moderate agreement in depth assessments but weak agreement in speed and recoil compared with QCPR performance metrics. Previous studies have revealed that certified instructors frequently face challenges in accurately assessing inadequate chest compression depth during CPR training sessions [4]. Consistent with these findings, our own research demonstrated that while instructors can generally evaluate chest compression depth with acceptable precision, their ability to accurately judge recoil and compression rates remains less reliable. This issue persisted despite the use of different manikins in comparative studies, leading to consistent results across diverse training tools. This consistency underscores a broader issue: the reliance on visual assessment for determining the quality of CPR is notably unreliable. This uncertainty is further complicated by the variety of technologies and mechanisms present in different manikins, which can significantly impact what instructors observe and, subsequently, their evaluations. Instructors may certify trainees who lack proficiency in critical CPR skills due to challenges in accurately assessing these techniques. Such deficiencies in training can pose serious risks in emergencies, as inadequately trained individuals may not perform CPR effectively, jeopardizing patient outcomes.

The performance discrepancies observed across different types of manikins emphasize the urgent need for standardization in CPR training equipment. Establishing uniform standards would ensure more consistent and precise evaluations by instructors, regardless of the training setting or equipment used. Furthermore, the implementation of CPR feedback devices has been suggested as a means to increase the accuracy of these assessments [10]. These devices can provide real-time, objective data on the quality of chest compressions, offering instructors clear feedback and reducing the subjective nature of manual evaluations.

Another crucial observation from this study is the significant correlation between the depth of chest compression and the rate of BLS instructor observation. Prior research indicates that the duration of dynamic visual stimuli—such as moving hands during CPR—is perceived as longer than that of static stimuli of the same duration [16]. This perceptual distortion can influence CPR training outcomes, as instructors often perceive greater compression depth when the rate of compression increases, a perception likely driven by the increased visual motion intensity associated with faster movements.

This phenomenon highlights the reduction in actual compression depth as the rate increases, which may lead to discrepancies between the assessments of the BLS instructor and the QCPR [17]. This is supported by theories suggesting that the perceived intensity of visual motion increases with speed. As a result, instructors may inaccurately judge the correct depth and speed of chest compressions, leading to a higher occurrence of false positives in CPR skill evaluations.’ [4–6, 18]. Such inaccuracies pose severe risks as trainees deemed proficient may not meet essential skill benchmarks, potentially compromising patient safety in real-life resuscitation situations.

The American Heart Association (AHA) recommends the incorporation of real-time feedback mechanisms and structured post-resuscitation debriefings to counter these issues [19]. The implementation of real-time, objective feedback mechanisms and post-scenario quantitative debriefing via objective metrics has shown promise in addressing deficiencies in CPR training while simultaneously reducing the cognitive burden on instructors. Research indicates that such real-time feedback systems can significantly enhance performance during CPR training sessions [10, 20, 21]. Furthermore, a variety of tools, including high-fidelity manikins, metronomes, and smartphones [22], have been effectively employed to assess and improve the quality of chest compressions in various training environments. Moreover, the use of peer feedback within training contexts has shown considerable promise. It has been deemed both feasible and advantageous in refining the facilitation skills of tutors [8]. This approach not only fosters a collaborative learning environment but also encourages continuous professional development among instructors, enhancing their ability to deliver high-quality training. By integrating peer feedback mechanisms, training programs can leverage collective expertise, improve instructional techniques, and ultimately increase the standard of CPR education provided. Furthermore, a previous study highlighted the effectiveness of peer video recording feedback (PVF) over traditional verbal feedback (TVF) in enhancing CPR skills acquisition and retention among medical students [23]. The findings indicated that the PVF group achieved significantly better results in terms of overall scores, compression depth, and chest recoil. Importantly, these advantages could also be beneficial in low-resource areas, suggesting that PVF might be an effective training enhancement in settings with limited medical training facilities. These innovations provide instructors with precise, actionable data that can lead to more effective teaching strategies and improved learning outcomes.

## Limitations

This study has several limitations that should be considered when interpreting the results. First, the analysis was based on three cycles of chest compressions, totaling 90 compressions. This corresponds to approximately 40–50 s of compressions, which is a relatively short observation period and may not fully capture the variability in CPR quality over the longer durations typically recommended during resuscitation.

Second, instead of using actual measured data, we employed a converted grading system for evaluations, which may have widened the discrepancies between the BLS instructor assessments and the QCPR device readings. This conversion could distort the accuracy of comparisons.

Third, the use of high-fidelity simulation manikins, while beneficial for standardized training, may not fully replicate the dynamics of chest compressions on human subjects. Besides, there might exist the system errors with the calibration of the manikin used in the study to reflect the precise depths measurement. This limitation suggests that the findings might not be entirely applicable to real-world CPR situations.

At last, the resuscitation manikin used in this study is a commercially available model widely used in resuscitation science research. Although this ensures that our findings are relevant to common training protocols, it is crucial to recognize the potential for measurement errors, particularly in scenarios that involve vigorous chest compressions. Moreover, if a different type of manikin was used, the results could have varied, indicating that the choice of equipment can significantly influence study outcomes.

## Conclusions

A comparison of instructor assessments with manikin data revealed that certified BLS instructors demonstrated a limited ability to assess CPR skills accurately. Instructors’ visual assessment of chest compression quality reveals a significant influence of the compression rate on the recognition of compression depth. To improve the accuracy of CPR skill assessments, instructors must be educated about this bias and trained to recognize and adjust for its impact [24, 25].

Furthermore, the incorporation of CPR feedback devices is suggested as a viable method to ensure the consistency of CPR quality. These devices can offer real-time, objective feedback that helps align instructor assessments with standardized performance metrics.

Additionally, employing methods of self-observation and peer feedback within the training framework can substantially improve the facilitation skills of tutors. These educational adjustments are vital for properly equipping health care professionals with the necessary skills to perform life-saving interventions both accurately and efficiently, ensuring that they are well prepared to handle real-world emergencies effectively.

## Data Availability

The data utilized in this study can be accessed upon reasonable request to the corresponding author.

## Abbreviations

OHCA: Out-of-hospital cardiac arrest
CPR: Cardiopulmonary resuscitation
AEDs: Automated external defibrillators
BLS: Basic Life Support
ERC: European Resuscitation Council
QCPR: Quantitative CPR
